# The Quantum Casimir Effect May Be a Universal Force Organizing the Bilayer Structure of the Cell Membrane

**DOI:** 10.1007/s00232-013-9544-9

**Published:** 2013-04-24

**Authors:** Piotr H. Pawlowski, Piotr Zielenkiewicz

**Affiliations:** 1 Institute of Biochemistry and Biophysics, Polish Academy of Sciences, PAS, Pawinskiego 5a, 02-106 Warszawa, Poland; 2 Plant Molecular Biology Laboratory, Warsaw University, Warsaw, Poland

**Keywords:** Cell membrane, Lipid bilayer, Casimir effect

## Abstract

A mathematic–physical model of the interaction between cell membrane bilayer leaflets is proposed based on the Casimir effect in dielectrics. This model explains why the layers of a lipid membrane gently slide one past another rather than penetrate each other. The presented model reveals the dependence of variations in the free energy of the system on the membrane thickness. This function is characterized by the two close minima corresponding to the different levels of interdigitation of the lipids from neighbor layers. The energy barrier of the compressing transition between the predicted minima is estimated to be 5.7 kT/lipid, and the return energy is estimated to be 3.1 kT/lipid. The proposed model enables estimation of the value of the membrane elastic thickness modulus of compressibility, which is 1.7 × 10^9^ N/m^2^, and the value of the interlayer friction coefficient, which is 1.9 × 10^8^ Ns/m^3^.

## Introduction

Casimir-Polder (Casimir 1948; Casimir and Polder 1948) forces are universal physical forces arising from a quantized field. They act even between two uncharged metallic plates in a vacuum, placed a few micrometers apart, without any external electromagnetic field. The idea that Casimir forces may play an important role in different biomembrane systems is quite new. Based on the fundamental work of Lifshitz (1956) dealing with retarded van der Waals forces between macroscopic bodies, first it was applied to the formation of cellular “rouleaux” (Bradonjić et al. 2009) and confined biomembranes (El Hasnaoui et al. 2010). In a natural manner, it supplements the theory of nonretarded van der Waals interactions in a lipid–water system (Parsegian and Ninham 1970), sometimes offering an interesting counterproposal.

Thus, forces related to the zero-point energy of quantum fluctuations may play an important role in biology, and the analysis of these forces offers a new view into biological phenomena at the cellular level. Herein, we propose a simple second-quantized explanation for the fact that cell membrane bilayer leaflets slip (Otter and Shkulipa 2007) past one another, rather than penetrate each other. This membrane feature is of great importance as it determines the anisotropy of the membrane’s rheological properties. The relative freedom of movement of molecules along membrane leaflets and the relative restriction of displacement in the transverse direction account for the great lateral fluidity and the small perpendicular compressibility of a membrane (Evans and Hochmuth 1978). These dual mechanical properties, both fluid-like and solid-like, have an impact on the structure and function of the proteins embedded in the lipid bilayer matrix (Andersen and Koeppe 2007). This impact finally determines the status of the cell membrane as an active barrier, a natural organizer and an important participant in all processes of life.

The proposed model of the interaction of the cell membrane bilayer leaflets considers the membrane interior to be a three-layer dielectric sandwich. The free energy of the system is related to the electromagnetic field excitations in the ground state. The free energy depends on the varying thickness of the central layer, where lipid chains penetrate the opposite layers and where the density of lipid chains, and the dielectric permittivity, varies in space. The theoretical values of the two energy minima, the membrane elastic thickness modulus of compressibility and the interlayer friction coefficient, were set according to this model.

## The Model

**Fig. 1:**
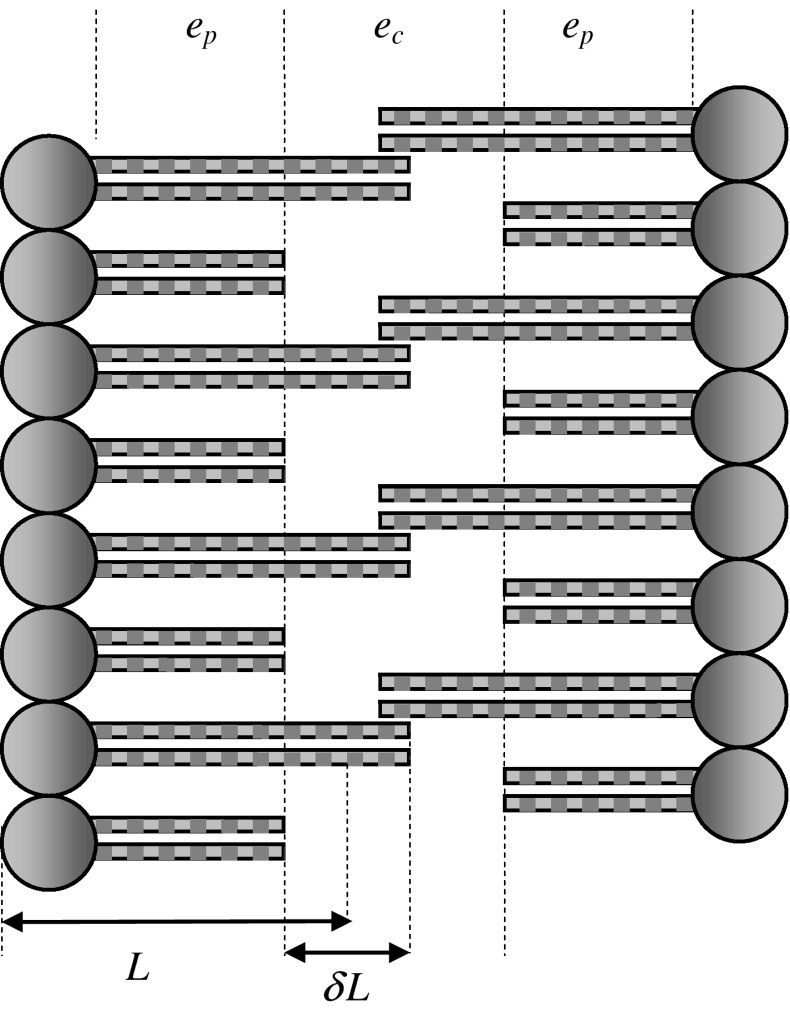
Three-layer dielectric sandwich model of a cell membrane. Only the longest and shortest lipids are shown. *L* average lipid length, *δL* width of the lipid length range, *ε*_c_ dielectric constant of the central region, *ε*_p_ dielectric constant of the peripheral region

The cell membrane interior was considered to be a three-layer dielectric sandwich, which consists of parallel slabs as in Fig. 1. The two lateral peripheral regions of this system are fully occupied by all the lipids of the local leaflet. The remaining central region contains the hydrocarbon tails that penetrate from the neighboring layers. The dielectric constant, *ε*_c_, of this central layer differs from the dielectric constant, *ε*_p_, of the other parts of the membrane. We assumed that the length of the lipids in each leaflet may vary within a narrow range, *L* ± *δL/*2, where *L* represents the average lipid length and *δL* represents the width of the length distribution. The distribution of the lipid lengths was assumed to be uniform. Thus, the perpendicular cross section of a membrane bilayer matrix resembles “the two overlapping combs with some broken teeth.”

**Fig. 2:**
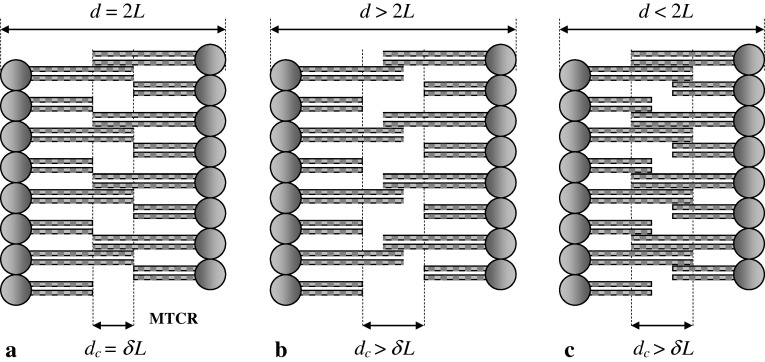
Unidirectional variations in the thickness of the central region of the membrane. Only the longest and shortest lipids are shown. **a** Configuration MTCR. Total membrane thickness *d* = 2*L*. Central region thickness *d*_c_ = *δL*. **b** Bilayer leaflets farther apart than in configuration MTCR. Total membrane thickness *d* *>* 2*L*. Central region thickness *d*_c_ *>* *δL*. **c** Bilayer leaflets closer together than in configuration MTCR. Total membrane thickness *d* *<* 2*L*. Central region thickness *d*_c_ *>* *δL*. *L* average lipid length, *δL* width of the lipid length range, *MTCR* minimal thickness of the central region

The thickness of the central region, *d*_c_, falls to a minimum value equal to *δL* in the configuration in which the total membrane thickness, *d*, equals 2*L* (Fig. 2a). This configuration we called “configuration MTCR” (minimal thickness of the central region). To clarify further considerations, configuration MTCR was treated as the reference configuration. In this configuration, *d*_c_ may increase both with an increase (Fig. 2b) and with a decrease (Fig. 2c) in the total membrane thickness. Thus, the possible variation in the thickness of the central region in configuration MTCR is unidirectional.

For convenience, we introduced the variable *x* to describe the difference between the actual total membrane thickness and the thickness of the membrane in configuration MTCR; that is, *x* = *d−*2*L.* Then to formalize the description of the central region, one may write *d*_c_ = *δL* + |*x*|, where |*x*| denotes the absolute value.

The dielectric constant, *ε*_c_, was assumed to vary in space within the central region due to variations in the lipid density. Variations along the direction perpendicular to the membrane plane were postulated. At first approximation, *ε*_c_ was simply characterized by the spatial average 〈*ε*_c_〉. In general, when the total membrane thickness differs from the thickness of the reference configuration by *x*, the average 〈*ε*_c_〉 changes as described by the following formula:

1 $$ \langle \varepsilon_{\text{c}} \rangle = \left\{ {\begin{array}{*{20}c} {1 + \left( {\varepsilon_{\text{p}} - 1} \right)\left( {1 + \frac{|x|/\delta L}{1 + |x|/\delta L}} \right)} & {x < 0} \\ {\varepsilon_{\text{p}} } & {x = 0} \\ {1 + \left( {\varepsilon_{\text{p}} - 1} \right)\frac{1}{1 + x/\delta L}} & {x > 0} \\ \end{array} } \right. $$

For details, see Appendix.

**Fig. 3:**
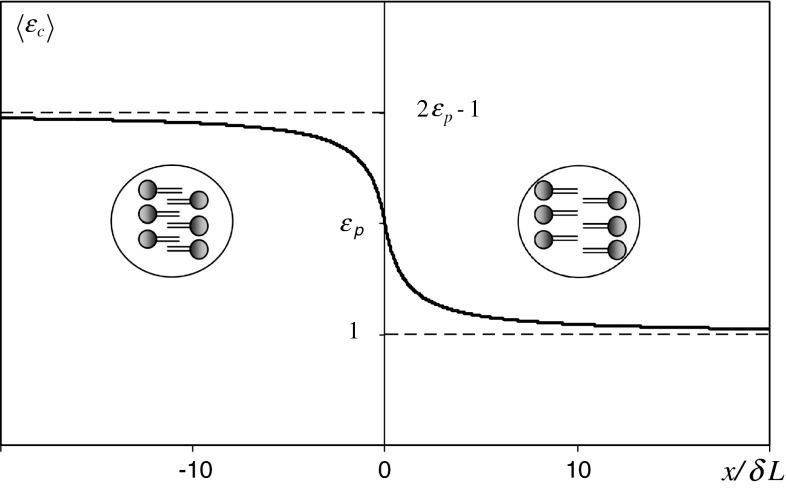
The space average of the dielectric constant in the central region, 〈*ε*_c_〉, as a function of the ratio *x*/*δL. x* the difference between the total membrane thickness and the membrane thickness in configuration MTCR, *δL* width of the lipid length range, *ε*_p_ dielectric constant in peripheral regions of the membrane

The plot of Eq. 1 indicates (Fig. 3) that the average 〈*ε*_c_〉 increases with the compression of the membrane thickness (*x* < 0) and decreases with membrane thickness extension (*x* > 0). For the membrane in the configuration MTCR (*x* = 0), 〈*ε*_c_〉 strictly equals *ε*_p_.

Quantum electrodynamic considerations reveal that two dielectric plates separated by a dielectric medium may be attracted as a result of the decrease in the zero-point energy of the quantum electromagnetic field excitations (Srivastava et al. 1985). In the case of the cell membrane, by approximating the dielectric constant of the central region with the average value 〈*ε*_c_〉, the free energy, *F*, of the field per unit area may be described by the following equation (Bradonjić et al. 2009):

2 $$ F = - \frac{{\pi^{2} }}{720}\left( {\frac{{\varepsilon_{\text{p}} - \langle \varepsilon_{\text{c}} \rangle }}{{\varepsilon_{\text{p}} + \langle \varepsilon_{\text{c}} \rangle }}} \right)^{2} \frac{\hbar c}{{\sqrt { \langle \varepsilon_{\text{c}} \rangle } \left( {\delta L} \right)^{ 3} \left( {1 + |x|/\delta L} \right)^{ 3} \, }} $$

where $\hbar$ is the reduced Planck constant and *c* is the speed of light in a vacuum; the dependence of 〈*ε*_c_〉 on *x* is described by Eq. 1.

## Results

**Fig. 4:**
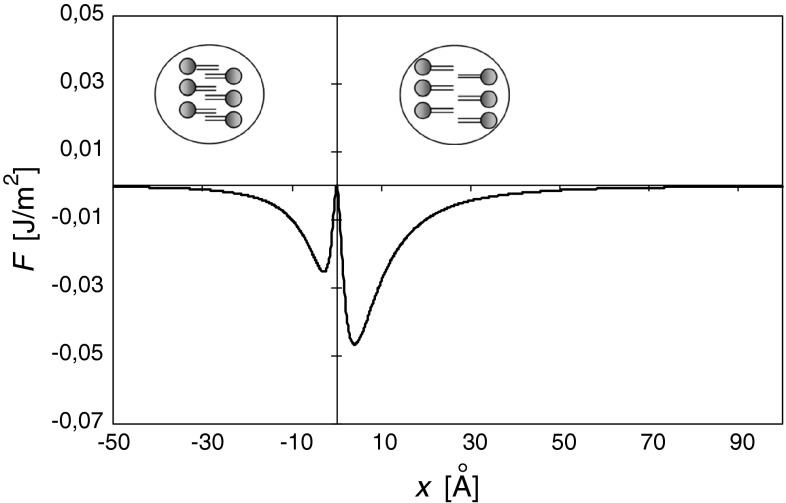
The free energy, *F*, of the field per unit area as a function of a change, *x*, in the membrane thickness relative to the thickness of the MTCR membrane

Equations 1 and 2 show that, for ideally flat leaflets (*δL* = 0), Casimir forces couple membrane layers at zero distance (*x* = 0), with the free energy tending to negative infinity. More reasonable calculations (δ*L* = 5 Å, *ε*_p_ = 2), taking into account the heterogeneity of the lipid lengths (Blaurock 1982) and specifying the dielectric constant of the peripheral regions of the membrane (Huang and Levitt 1977) were also performed. These calculations indicate (Fig. 4) that there are two finite minima of free energy, both for the membrane in configurations close to the MTCR. The first (lower) minimum (*F* = −4.68 × 10^−2^ J/m^2^) is for leaflets being slightly separated (*x* = 4.0 Å), and the second one (*F* = −2.54 × 10^−2^ J/m^2^) is for leaflets being moderately pushed together (*x* = −2.9 Å). According to the model, in both cases, the thickness of the central region of the membrane (*d*_c_ = 9.0 and *d*_c_ = 7.9 Å) is larger than the assumed thickness in the configuration MTCR (*d*_c_ = 5 Å).

## Discussion

**Fig. 5:**
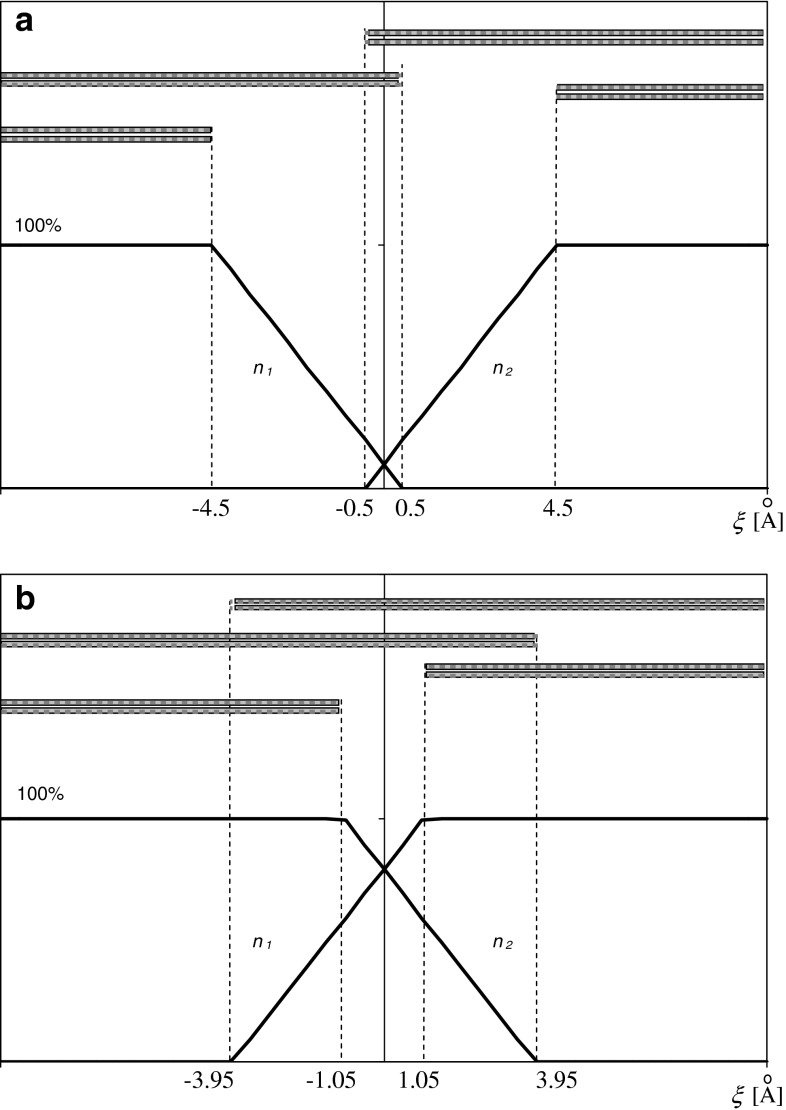
The area densities *n*_1_ and *n*_2_ of lipids belonging to a given leaflet as a function of a distance, *ξ*, from the membrane midplane. The range occupied by the longest and shortest lipids is shown above. **a** For the first minimum (lower) of free energy, *x* = 4 Å. **b** For the second minimum (higher) of free energy, *x* = −2.9 Å

We assumed that the central region of the membrane may be treated as a kind of leaflet interface. One interesting question is how the interface is stabilized to avoid leaflet penetration and sticking. The proposed model may offer a simple explanation based on the Casimir effect in dielectrics. At reasonable values of the applied parameters (see “Results” section), the model predicts two free energy minima. For the membrane organized in the lower energy minimum (*x* = 4 Å), its leaflets are separated by 4 Å greater than in configuration MTCR. At the assumed heterogeneity of the lipid lengths (*δL* = 5 Å), this result means that 20 % of lipids (1−*x*/*δL*) penetrate into the neighboring leaflet (Fig. 5a) but by no more than 1 Å (*δL−x*).

According to the results presented in Fig. 4, transition into the second (higher) minimum (*x* = –2.9 Å) requires approximately 5.7 kT/lipid (calculated for *T* = 300 K and area per lipid *a* = 50 Å^2^). In this minimum energy configuration, the membrane leaflets are in closer contact. All lipids penetrate the opposite layer but no deeper than 7.9 Å (Fig. 5b). The return to the other minimum requires 3.1 kT/lipid.

The revealed characteristic energies are several times higher than those estimated assuming the average tension γ (0.003 mN/m) of living cells (Blanchard and Rauch 2012). The result γa ≪ kT indicates that the Casimir effect may be an important contributor to the membrane dynamics, along with the hydrophobic effect.

To study the mechanical properties of the presented model membrane, the elastic thickness modulus of compressibility, *k*, was numerically estimated using a differential second derivative of the free energy at the first minimum (*k* = *d F″*). Assuming a bilayer thickness of *d* = 5 nm (Kuchel and Ralston 1998), *k* = 1.7 × 10^9^ N/m^2^; i.e., this estimated value of *k* is of the same order as that measured using volume dilatometry of lipid bilayers (Srinivasan et al. 1974). This result indicates that the central region of the cell membrane resembles an “incompressible” core that does not allow lipid interleaflet penetration.

The results of the presented model also enable estimation of the value of the interlayer friction coefficient, *f* (friction force per unit area and unit velocity). Approximating the end of the lipid penetrating the neighbor leaflet with a hemisphere of a radius *r* that experiences the action of the Stokes force from the liquid passing with the velocity 2*v,* one may obtain *f* = 6π*ηr*/*a,* where *η* is the membrane shear viscosity. Taking the typical value of viscosity *η* = 0.1 Ns/m^2^ estimated from the diffusion coefficient of membrane-spanning proteins in phospholipid bilayers (Waugh 1982) and assuming that the membrane is in the first minimum with *r* = 0.5 Å, *f* = 1.9 × 10^8^ Ns/m^3^, which is within the range of reported experimental values (Shkulipa et al. 2005; Otter and Shkulipa 2007). For the second minimum, one may expect an eightfold higher value because of a deeper penetration.

According to the model, a membrane bilayer in a basic state (lower minimum) should possess relatively small interleaflet friction. The probability that, due to thermal fluctuations, some regions of the membrane reach second minimum is relatively small.

**Fig. 6:**
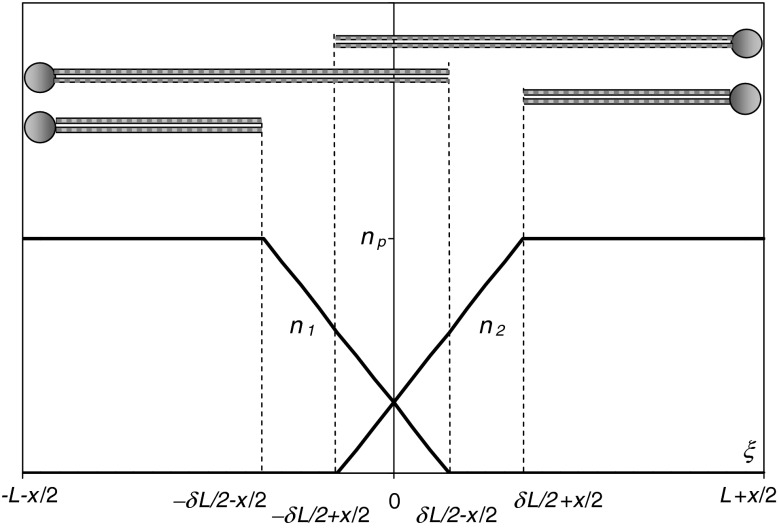
The area densities *n*_1_ and *n*_2_ of lipids belonging to a given leaflet as a function of the distance, *ξ*, from the membrane midplane. The range occupied by the longest and shortest lipids is shown above. *n*_p_ total number of lipids belonging to a given leaflet per unit surface area, *L* average lipid length, *δL* width of the lipid length range, *x* *=* *d* *−* 2*L*, where *d* is the total membrane thickness

At first glance, one may worry about some physical and mathematical problems with the proposed approach. It is obvious that some points require additional discussion, especially phenomena at a physical level neglected in our model. First of all, is it justified to assume that the lipids of opposite leaflets interpenetrate each other at all? Steric interactions seem to be the dominant suppressor of interdigitation. However, a spontaneous or induced interdigitated phase of bilayers consisting of double-tail lipids was confirmed in computer simulation and differential scanning calorimetry experiments (Kranenburg 2004; Kranenburg et al. 2004; Mavromoustakos et al. 2011). Moreover, a simple estimation below shows that the possible steric effect is not energetically dominant, as one may expect. Let us assume that during interdigitation the part *p* of lipids is compressed and their length decreases by an assumed certain value, *λ*. Let us also assume that each of two lipid acyl chains contains the number *b* of C–C bonds characterized by a certain bond stiffness, *g*. Then, elastic deformation energy per lipid molecule may be calculated as *pgλ* ^*2*^ */b*. For typical conditions, *p* = 1/10 (meaning that 50 % of penetrating lipids in the lower minimum are compressed), *g* = 100 N/m, *λ* = 0.5 Ǻ and *b* = 15; this energy equals 0.8 *k*_*B*_ *T*. These are only 7 % of the predicted value of the energy barrier in a lower minimum and, as such, may be neglected at first approximation. The next question is, how much does the derived shape of the free energy, *F* (Fig. 4), depend on the specific way that the interpenetration occurs? It was assumed that the distribution of lipid lengths was uniform and, in this way, interdigitation varied linearly with a distance from the membrane midplane (Figs. 5, 6). What will change if we assume the more spectacular variation? In extreme cases, when a single-point distribution (*δL* = 0) is assumed, one infinite minimum of energy at midplane will be obtained and the energy will increase with distance, like –1/|*x*|^3^. Thus, narrowing the distribution lowers minima and approaches them together. It should be stressed that the parameters 〈*ε*_c_ *〉*, *ε*_p_ and *δL* in Eq. 2 may also effectively describe a more diverse system.

Applied formula for the free energy per unit area, Eq. 2, assuming *ε*_p_ → ∞ and 〈*ε*_c_〉 → ∞, gives the famous Casimir result for the energy of attraction between ideal mirrors. This energy is a result of the change in the zero-point energy of an empty quantum vacuum. From the other hand, it is well known that real cell membranes are under the permanent influence of electrostatic interactions. Assuming a natural transmembrane electric potential *V* = 100 MV, a membrane thickness *d*_m_ = 10^−8^, an effective membrane dielectric constant *ε*_m_ = 2 and a vacuum permittivity *ε*_0_ = 10^−11^ [F/m], it is easy to estimate the area density of the energy of an electric field, 0.5*ε*_*0*_ *ε*_m_ (*V* ^2^ */d*_m_). It is equal to 10^−5^ J/m^2^ . This result is three orders less than the energy of the considered Casimir effect.

It is necessary to underline that the discussed formula is correct only within certain assumed constraints. One of them, zero temperature approximation (Landau and Lifshitz 1960) may be justified for considered conditions (*k*_*B*_ *T* *≪* *ħc/d*_c_). The second assumption, i.e., the same dielectric constant at all frequencies, is formally valid for ideal dielectrics or in the case of large separations. However, for values of constant permittivity not so far from unity (vacuum value), possible error for small distances is disregarded. Moreover, the bulk term in energy is disregarded, which may be dominant for large separations. As the ratio of energies of attraction of water slabs and lipid slabs (both estimated using Eq. 2) is as small as 1:10, for the sake of simplicity, the influence of water outside the membrane was not considered. Anisotropy in the dielectric constant is also beyond the scope of this article, and the model for the dielectric constant in the region of interdigitation is very simple, based on a linear superposition of dielectric constants in terms of the effective densities of tails.

We realize that real membranes, especially cell membranes, are obviously complex, heterogeneous, nonideal dielectrics with complicated frequency responses and real conductivity. They are certainly not perfectly plain and smooth plates. Despite the above simplifications, the general predictions of our model, i.e., the magnitude of the free energy and the existence of two energy minima, seem to be still reasonable and wait for more detailed further investigation and confirmation. A lateral Casimir effect for corrugated planes or nonretarded local pairwise van der Waals forces might provide a better description of the physics there and provide a more accurate description. For example, the last one can replace an inverse-cube law, describing variation in energy with distance, by an inverse-square law. Using this method, a simple estimation of energy of so-called hydrophobic bonding, at a Hamaker function equal to 7 × 10^−21^ J and a distance of 50 Ǻ (Parsegian and Ninham 1970), gives the value of the density of free energy close to 10^−5^ J/m^2^. This quantity is three orders less than the energy of lipid–lipid interactions and two orders less than the energy of water–water interactions estimated in our model. We think that future numerical brute-force simulations might make an important contribution toward a better understanding of the mentioned discrepancy.

In light of these findings, it is evident that the Casimir effect may play an important role in many biological phenomena and may be a universal force that organizes the tensegrity structure of biological systems. Some scientists might expect spectacular “levitation” forces between the leaflets, but it appears that there is instead a “quantum trap” preventing membrane leaflet interdigitation and collapse as well as maintaining a significant gap between leaflets, which leaves molecules a plane with freedom of movement. Even without taking into account interlayer lipid collisions, hydrophobic interactions and the stabilizing role of proteins, Casimir forces may prevent lipid chain mishmash or molecular escape. This hypothesis appears to be fruitful and is worth experimental verification.

## Appendix

### The Average Value of the Dielectric Constant in the Central Region of a Lipid Bilayer

Let variable *ξ* represent the distance from the membrane midplane; then, the central region may be defined as the membrane layer within the range $- \delta L/ 2- \left| x \right|/ 2 \le \xi \le \delta L/ 2 + \left| x \right|/ 2$. Here, *δL* represents the width of the distribution of lipid lengths, and *x* = *d* − 2*L*, where *d* is the total membrane thickness and *L* is the average lipid length. In general, *x* may be positive (thickness expansion), negative (thickness compression) or zero (configuration MTCR) and falls within the range *x* > *δL* − 2*L*. Assuming that the dielectric constant *ε*_c_ in the central region varies locally with *ξ*, *ε*_c_ depends on the number of lipids, *n*, that are passing through the unit area of plane *ξ* *=* constant. This dependence may be described as

3 $$ \varepsilon_{\text{c}} = 1 + (\varepsilon_{\text{p}} - 1)\frac{n}{{n_{\text{p}} }} $$

where *ε*_p_ is the dielectric constant of the peripheral region of the membrane fully occupied only by the lipids belonging to a given leaflet and *n*_p_ is the total number of lipids belonging to a given leaflet per unit surface area.

The area density *n* of lipids at distance *ξ* can be described by the equation

4 $$ n = n_{1} + n_{2} $$

where *n*_1_ and *n*_2_ are area densities of lipids belonging to a given leaflet.

For a uniform distribution of lipid lengths, the constituent densities *n*_1_ and *n*_2_ (Fig. 6) can be described as follows:

5 $$ n_{1} = \left\{ {\begin{array}{*{20}c} {n_{\text{p}} } & { - L - x/2 \le \xi < - \delta L/2 - x/2} \\ {\frac{{n_{\text{p}} }}{2}\left( {1 - \frac{\xi + x/2}{\delta L/2}} \right)} & { - \delta L/2 - x/2 \le \xi \le \delta L/2 - x/2} \\ 0 & {\delta L/2 - x/2 < \xi \le L + x/2} \\ \end{array} } \right. $$

6 $$ n_{2} = \left\{ {\begin{array}{*{20}c} 0 & { - L - x/2 \le \xi < - \delta L/2 + x/2} \\ {\frac{{n_{\text{p}} }}{2}\left( {1 + \frac{\xi - x/2}{\delta L/2}} \right)} & { - \delta L/2 + x/2 \le \xi \le \delta L/2 + x/2} \\ {n_{\text{p}} } & {\delta L/2 + x/2 < \xi \le L + x/2} \\ \end{array} } \right. $$

Independently of the sign of *x*, the central region consists of three layers:

7 $$ \left\{ {\begin{array}{*{20}c} { - \delta L / 2- \left| x \right|/2 \le \xi < - \delta L / 2+ \left| x \right|/2} \hfill \\ { - \delta L / 2+ \left| x \right|/2 \le \xi \le \delta L / 2- \left| x \right|/2} \hfill \\ {\delta L / 2- \left| x \right|/2 < \xi \le \delta L / 2+ \left| x \right|/2} \hfill \\ \end{array} } \right. $$

Calculating the sum in Eq. 4, with the help of Eqs. 5, 6 in respective layers, depending on the sign of *x,* one can obtain:

8 $$ n = \left\{ {\begin{array}{*{20}c} {n_{\text{p}} + \frac{{n_{\text{p}} }}{2}\left( {1 + \frac{\xi - x/2}{\delta L/2}} \right)} \hfill & { - \delta L / 2- \left| x \right|/2 \le \xi < - \delta L / 2+ \left| x \right|/2} \hfill & {} \hfill \\ {n_{\text{p}} \left( {1 - \frac{x}{\delta L}} \right)} \hfill & { - \delta L / 2+ \left| x \right|/2 \le \xi \le \delta L / 2- \left| x \right|/2} \hfill & {x < 0} \hfill \\ {n_{\text{p}} + \frac{{n_{\text{p}} }}{2}\left( {1 - \frac{\xi + x/2}{\delta L/2}} \right)} \hfill & {\delta L / 2- \left| x \right|/2 < \xi \le \delta L / 2+ \left| x \right|/2} \hfill & {} \hfill \\ \end{array} } \right. $$

9 $$ n = \begin{array}{*{20}c} {n_{\text{p}} } \hfill & { - \delta L / 2\le \xi \le \delta L / 2} \hfill & {x = 0} \hfill \\ \end{array} $$

10 $$ n = \left\{ {\begin{array}{*{20}c} {\frac{{n_{\text{p}} }}{2}\left( {1 - \frac{\xi + x/2}{\delta L/2}} \right)} \hfill & { - \delta L / 2- x/2 \le \xi < - \delta L / 2+ x/2} \hfill & {} \hfill \\ {n_{\text{p}} \left( {1 - \frac{x}{\delta L}} \right)} \hfill & { - \delta L / 2+ x/2 \le \xi \le \delta L / 2- x/2} \hfill & {x > 0} \hfill \\ {\frac{{n_{\text{p}} }}{2}\left( {1 + \frac{\xi - x/2}{\delta L/2}} \right)} \hfill & {\delta L / 2- x/2 < \xi \le \delta L / 2+ x/2} \hfill & {} \hfill \\ \end{array} } \right. $$

The space average of *n* in the range $- \delta L/ 2- \left| x \right|/ 2 \le \xi \le \delta L/ 2 + \left| x \right|/ 2$ is calculated as

11 $$ \langle n \rangle = \frac{{\int\limits_{ - \delta L / 2- \left| x \right|/2}^{\delta L / 2+ \left| x \right|/2} {nd\xi } }}{\delta L + \left| x \right|} $$

and according to Eqs. 8, 9 and 10 equals

12 $$ \langle n \rangle = \left\{ {\begin{array}{*{20}c} {n_{\text{p}} } \hfill {\left( {1 + \frac{\left| x \right|/\delta L}{1 + \left| x \right|/\delta L}} \right)}& {} \hfill & {x < 0} \hfill \\ {n_{\text{p}} } \hfill & {} \hfill & {x = 0} \hfill \\ {n_{\text{p}} \frac{1}{1 + x/\delta L}} \hfill & {} \hfill & {x > 0} \hfill \\ \end{array} } \right. $$

Then, the space average of the dielectric constant *ε*_*c*_ according to Eqs. 3 and 12 can be described as

13 $$ \langle \varepsilon_{c} \rangle = \left\{ {\begin{array}{*{20}c} {1 + \left( {\varepsilon_{\text{p}} - 1} \right)} {\left( {1 + \frac{|x|/\delta L}{1 + |x|/\delta L}} \right)} & {} \hfill & {x < 0} \hfill \\ {\varepsilon_{\text{p}} } \hfill & {} \hfill & {x = 0} \hfill \\ {1 + \left( {\varepsilon_{\text{p}} - 1} \right)\frac{1}{1 + x/\delta L}} \hfill & {} \hfill & {x > 0} \hfill \\ \end{array} } \right. $$
